# Supplementary material for: Evaluation of the Links between Lamb Feed Efficiency and Rumen and Plasma Metabolomic Data
Source: Metabolites. 2022 Mar 29;12(4):304. doi: 10.3390/metabo12040304 (PMC9029153; doi:10.3390/metabo12040304)
Supplement: Supplementary file 1 [file metabolites-12-00304-s001.zip › metabolites-1605543-SI.pdf]

Table S1. Metabolites identified and quantified in the four biological fluid × diet combinations. For each metabolite, the number of samples in which it has been identified is expressed followed by the prevalence percentage between parentheses.

	Plasma		Rumen	
	CONC (n=274)	MIX (n=166)	CONC (n=273)	MIX (n=164)
	Number of samples in which the metabolite was identified (prevalence %)			
**Organic acids**				
Acetate	274 (100)	166 (100)	273 (100)	164 (100)
Azelaate			273 (100)	
Butyrate			273 (100)	164 (100)
Citrate	274 (100)	166 (100)		
Citraconate			232 (85.0)	76 (46.3)
Ethylmalonate			273 (100)	164 (100)
Gluconate		166 (100)		
Beta-hydroxyisovalerate	274 (100)		273 (100)	
3-hydroxybutyrate	274 (100)	166 (100)		
4-hydroxyphenylacetate			272 (100)	
Isobutyrate			273 (100)	164 (100)
Isovalerate				164 (100)
Lactate	274 (100)	166 (100)	253 (92.7)	
Malate	274 (100)		271 (99.3)	
2-oxobutyrate			253 (92.6)	
2-oxoglutarate			272 (99.6)	
2-oxoisovalerate	274 (100)			
Pantothenate	274 (100)			
3-phenylpropionate			273 (100)	164 (100)
Propionate			273 (100)	164 (100)
Pyroglutamate			269 (98.5)	
Pyruvate	264 (96.3)			
Sebacate				164 (100)
Succinate			248 (90.8)	164 (100)
Valerate			273 (100)	164 (100)
**Amino acids**				
N-acetylaspartate			273 (100)	164 (100)
Alanine			273 (100)	164 (100)

	2-aminobutyrate			273 (100)	
	Aspartate			273 (100)	
	N-Acetylglycine	274 (100)	166 (100)	267 (97.8)	
	Betaine	273 (99.6)	166 (100)		
	Citrulline	274 (100)			
	Creatine	274 (100)	166 (100)		
	Cystine	274 (100)	166 (100)	273 (100)	
	GABA			273 (100)	
	Glutamate	274 (100)		273 (100)	
	Glutamine	274 (100)			
	Glycine	274 (100)	166 (100)	273 (100)	164 (100)
	Isoleucine			273 (100)	
	Leucine	274 (100)	166 (100)	268 (98.1)	164 (100)
	Methionine			272 (99.6)	
	3-methylxanthine			273 (100)	164 (100)
	Phenylalanine	274 (100)		273 (100)	
	Proline			273 (100)	
	Serine	274 (100)	166 (100)		
	Taurine	274 (100)			
	Threonine	274 (100)	165 (99.4)	273 (100)	164 (100)
	Tyrosine			273 (100)	
	Valine	274 (100)	166 (100)		
**Alcohols**					
	Choline chloride	265 (96.7)	161 (97.0)	273 (100)	
	Ethanolamine	265 (96.7)	166 (100)		
	4-Ethylphenol			273 (100)	
	Glycerol	274 (100)	166 (100)		
	Methanol	274 (100)	166 (100)	187 (68.5)	
	Myo-inositol	274 (100)			
	Phosphocholine	271 (98.9)	166 (100)		
**Other metabolites**					
	Cadaverine			269 (98.5)	164 (100)
	Carnitine	274 (100)	166 (100)		
	Creatinine	274 (100)	166 (100)		
	Dimethylsulfone	274 (100)	166 (100)	273 (100)	164 (100)

	Glucose	274 (100)	166 (100)	272 (99.6)	
	Levoglucosan			273 (100)	
	TMAO	259 (94.5)			
	Uracil	274 (100)		273 (100)	

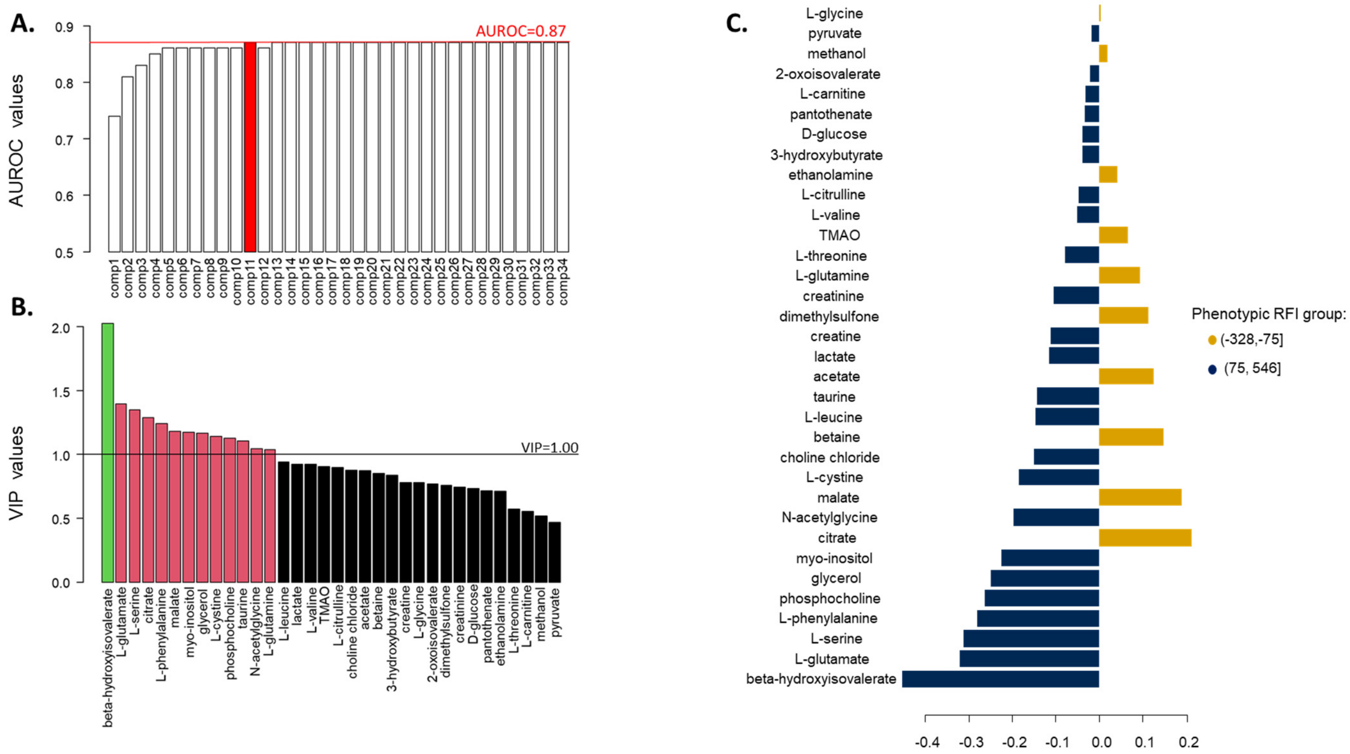

Figure S1. Discriminant analysis of the plasma metabolites measured during the CONC phase according to phenotypic RFI groups. Efficient animals are the ones that had a phenotypic RFI between −328 and −75 g/day and inefficient animals are the ones that had a phenotypic RFI between 75 and 546 g/day. (A) Accuracy of the PLS-DA models assessed using AUROC; red line marks the maximum AUROC value obtained with 11 components. (B) Selection of the variables contributing the most to the discriminant analysis using a VIP approach. (C) Loading values assigned to each metabolite on the first component of the PLS-DA model; gold and blue represent metabolites associated with efficient and inefficient animals, respectively.
